# Synthesis and investigation of SiO_2_-MgO coated MWCNTs and their potential application

**DOI:** 10.1038/s41598-019-51745-1

**Published:** 2019-10-22

**Authors:** Krisztian Nemeth, Nikolett Varro, Balazs Reti, Peter Berki, Balazs Adam, Karoly Belina, Klara Hernadi

**Affiliations:** 1 Department of Applied and Environmental Chemistry, University of Szeged, Rerrich Béla tér 1, HU-6720 Szeged, Hungary; 2 GAMF Faculty of Engineering and Computer Science, John von Neumann University, Izsáki út 10, HU-6000 Kecskemét, Hungary

**Keywords:** Synthesis and processing, Nanoparticles

## Abstract

In the present publication, multiwalled carbon nanotubes (MWCNT) coated with SiO_2_–MgO nanoparticles were successfully fabricated via sol–gel method to facilitate their incorporation into polymer matrices. Magnesium acetate tetrahydrate and tetraethyl orthosilicate were used as precursors. The coated MWCNTs were characterized by transmission electron microscopy (TEM), X–ray diffraction (XRD) and Raman spectroscopy methods. These investigation techniques verified the presence of the inorganic nanoparticles on the surface of MWCNTs. Surface coated MWCNTs were incorporated into polyamide (PA), polyethylene (PE) and polypropylene (PP) matrices via melt blending. Tensile test and differential scanning calorimetry (DSC) investigations were performed on SiO_2_–MgO/MWCNT polymer composites to study the reinforcement effect on the mechanical and thermal properties of the products. The obtained results indicate that depending on the type of polymer, the nanoparticles differently influenced the Young’s modulus of polymers. Generally, the results demonstrated that polymers treated with SiO_2_-MgO/MWCNT nanoparticles have higher modulus than neat polymers. DSC results showed that nanoparticles do not change the melting and crystallization behavior of PP significantly. According to the obtained results, coated MWCNTs are promising fillers to enhance mechanical properties of polymers.

## Introduction

Particulate-filled polymer composites are a widely researched field both in the academic and industrial area. The incorporation of different filler materials into thermoplastics – such as polyamide (PA), polyethylene (PE) or polypropylene (PP) – is a common practice in the polymer industry to reduce the production cost and improve the properties of products. Inorganic fillers are also applied to modify the properties of the raw polymer, such as the mechanical and electrical properties. Experiments with kaolin^1,2^, montmorillonite^3,4^, or talc^5–8^ filled polymers are well known. Talc is one of the most popular filler materials for thermoplastics, because of its low cost, softness and particulate morphology. Talc is a hydrous magnesium silicate, containing sheets of magnesium in octahedral coordination, sandwiched between sheets of silicon in tetrahedral coordination, which are held together by weak Van der Waals forces. The typical composition of talc is 63.5% SiO_2_, 31.7% MgO, and 4.8% H_2_O. The effect of fillers usually depends greatly on their morphology, particle size, interfacial interaction with matrices, particle size distribution and content^9^. To achieve the desired properties at lower filler loading, nanofillers are often used. Filler with higher surface area can contribute to more contact surface between the polymer matrix and filler. However, finer particles tend to agglomerate and this can cause disadvantageous effects on mechanical properties. Due to their unique properties^10^, MWCNTs have emerged as the most promising nanofiller candidate for polymer composites^11–15^ as of their discovery^16^. MWCNTs with rolled graphitic layers with remarkable mechanical^17–19^, thermal^20,21^ and electrical^22–24^ properties can be applied as reinforcement of fiber/matrix composites to achieve improved properties. In the last decades, the use of MWCNTs as filler in thermoplastics such as PE^25–27^, PP^28–31^ or polycarbonate^32–34^ has been widely studied. However, to take advantages of the reinforcement characteristics of MWCNTs, hindering factors have to be eliminated, such as the strong Van der Waals interaction between the MWCNTs and their poor surface wetting properties. Thus far, numerous methods have been carried out to treat the surface of MWCNTs, improving the better interaction between filler and polymer^35–37^. One possible solution to the above-mentioned problems is to coat the surface of MWCNTs with inorganic metal oxide layers. Many researches were concerned with the synthesis of oxide/MWCNT nanocomposites to apply them as photocatalysts^38–40^, gas sensors^41,42^, or for defluoridation of water^43^. The effects of silica coated MWCNT nanocomposites on the properties of polymer matrices are extensively studied^44–46^.

The joint application of two geometrically different fillers, such as MWCNT coated with inorganic nanoparticles may reduce the total filler content due to the co-supporting effect of the two fillers. These crystalline and fiber shaped filler materials together may open a promising path in the polymer industry to produce polymer composites at low filler content and low costs.

The main goal of this work was to synthetize silica and magnesia coated MWCNTs to get maximum guarantee of perfect dispersion and investigate their effect on the properties of polymers. Attempts on synthesizing SiO_2_-MgO coated MWCNT/polymer composites were not yet extensively investigated, thus this work might present useful information for synthesis and investigation in the area of polymer science.

## Experimental Details

### Chemicals

**Figure 1:**
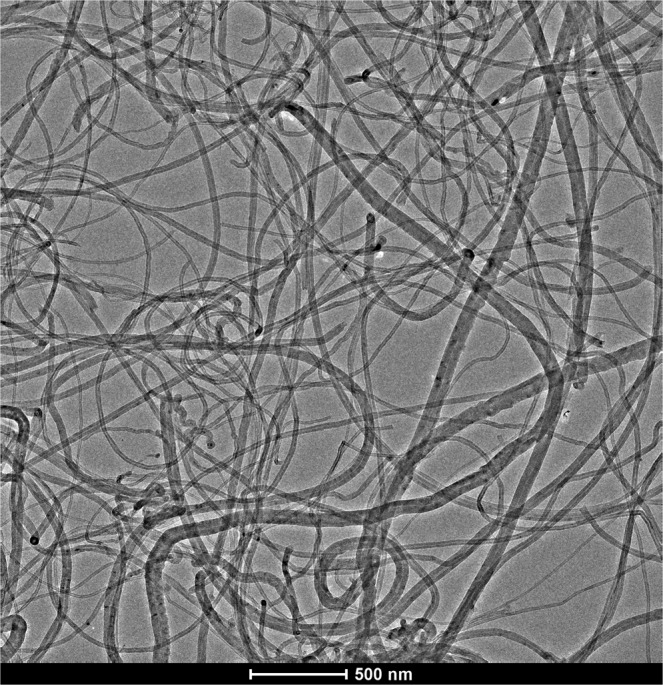
TEM image of pristine MWCNT.

All chemicals were of analytical grade. Magnesium acetate tetrahydrate [Mg(CH_3_COO)_2_ × 4H_2_O] and tetraethyl orthosilicate [Si(OC_2_H_5_)_4_] from Sigma-Aldrich, ethanol solvent from VWR, and ammonia solution from MolarChem were used without further purification for the preparation of the nanocomposite materials. The synthesis of MWCNTs was performed via catalytic chemical vapor deposition (CCVD) in a rotary oven at 720 °C applying Fe-Co/CaCO_3_ catalyst, nitrogen atmosphere and acetylene as carbon source. Using this synthesis process and catalyst, MWCNTs were selectively formed without amorphous carbon or catalyst particles encapsulated inside. The quality of the pristine MWCNT is crucial for both the nanocomposite and the polymer fabrication, and therefore, the purification of MWCNTs is an essential step in the beginning of experiments. The support and catalyst particles were removed by washing the MWCNT product with diluted hydrochloric acid, and afterwards with deionized water (MilliQ, 18.2 MΩ cm), until neutral pH was achieved. The average diameter of MWCNTs is about 20–60 nm, and the average lengths range from afew hundred nanometers to a couple of micrometers (Fig. 1).

PA (11) (melt flow rate [MFR – 230 °C/2.16 kg]: 3 g/10 mins, tensile strength at yield: 27 MPa) from Continental Inc., PE (8000F) (MFR: 6 g/10 mins, tensile strength at yield: 50 MPa) from TVK Inc. and PP (H 388F070359) (MFR: 9 g/10 mins, tensile strength at yield: 36 MPa) from TVK Inc. were applied as polymer matrices.

### Characterization methods

To verify and examine the formation of the SiO_2_–MgO layer on the surface of MWCNTs, TEM (FEI Tecnai G^2^ 20 X-TWIN) was used. For TEM investigations, a small amount of the sample was dispersed in ethanol via ultrasonication for 15 min then a small drop of the suspension was transferred onto a carbon film covered Cu TEM grid (Electron Microscopy Science, CF200-Cu). The crystal structure of the layer was investigated by XRD (Rigaku Miniflex II Diffractometer, angle range θ = 3–90) utilizing characteristic X-ray radiation (CuKα). The nanocomposites were also studied by Raman microscope with a built-in λ = 532 nm laser (Thermo Scientific DXR, spectrum acquisition time: 9 mins) in order to investigate whether the characteristic vibrations change of the species upon adding them to each other, which informs us about the presence of new interactions. The coated MWCNT/polymer composites were examined with scanning electron microscopy (SEM) with a cold field emission gun operating in the range of 5–15 kV (Hitachi S-4700 Type II FE-SEM). The crystallization and melting behavior of the coated MWCNT/polymer composites were investigated by differential scanning calorimetry (DSC). DSC measurements were carried out by a TA Instruments DMA Q800 device, applying a heating and cooling rate of 20 K/min, dynamic (20 mL/min) nitrogen atmosphere and indium for calibration. All samples having a weight of 3–5 mg were examined in a heat-cool-heat cycle. The degree of crystallinity of the polymer composites was calculated using the following formula:

$${X}_{c}=\frac{1}{1-wt \% }\frac{\Delta {H}_{m}}{\Delta {H}_{m}^{0}}\times 100$$

where *X*_*c*_ is the degree of crystallinity, wt% is the weight fraction of MWCNT, ΔH_*m*_ is the heat of fusion (enthalpy of melting) of nanocomposite, and ${\Delta H}_{{\rm{m}}}^{0}$ is the heat of fusion of 100% crystalline PP^47^

The mechanical properties of the composites were characterized by tensile tests, using an Instron 3366 tester at room temperature with a crosshead speed of 200 mm/min. In all the cases, three samples with the dimensions of 80 × 10 × 1 mm were measured and the mean values with standard deviation (SD) were calculated.

### Preparation of SiO_2_–MgO coated MWCNT nanocomposites

As the first step of synthesizing of SiO_2_–MgO coated MWCNT nanocomposites, oxidation was applied to produce carboxyl groups on the surface of MWCNTs. Carboxyl functionalized MWCNTs (f-MWCNTs) were prepared by ultrasonically dispersing the MWCNTs in 60 mL 3:1 mixture of HNO_3_:H_2_SO_4_ for 15 min. Afterwards, the mixture was stirred for 6 h at 65 °C, then MWCNTs were filtered in a membrane filtration apparatus (Durapore® VVPP membrane filter, pore size 0.1 µm) and washed with deionized water until pH = 7 was achieved. Finally, the functionalized and filtered MWCNTs were dried overnight at 70 °C. The SiO_2_–MgO/MWCNT nanocomposites were prepared by sol-gel technique, by varying the MWCNT loading. The initially predetermined amount of f-MWCNTs – varying between 0% and 5% by weight to calculated (5 g) total final weight of nanocomposites – was added to 60 cm^3^ ethanol and the suspension was sonicated for 30 min (solution A). Then, magnesia and silica precursors (molar ratio of Mg:Si = 3:4) were sonicated for 45 min in ethanol (Solution B). Afterwards, solution A was was vigorously stirred by a magnetic stirrer, and solution B was added in droplets (approximately 2 mL/min) to Solution A. To achieve the condensation reaction, the pH was set to 9–10 with 25% ammonia solution. The final mixture of Solution A and B was stirred overnight at room temperature to achieve the formation of a highly viscous gel. This gel was then placed in a drying furnace at 70 °C for 48 h to evaporate the solvent. After the drying process, white (samples without MWCNTs) and grayish-black (samples with MWCNTs) materials were obtained. Finally, the powder product was annealed in air at 400 °C for 4 h. The homogeneous white and grayish-black products were collected and pulverized into fine powder in an agate mortar.

### Preparation of SiO_2_–MgO coated MWCNT polymer composites

Due to the polar hydroxyl, carbonyl and amid functional groups, the PA matrix is capable of adsorbing moisture from the atmosphere, and for this reason, the drying process is a key step during the synthesis. Skipping this procedure may cause a damage on the surface of the polymer and due to the breakage of polymer chains, caused by hydrolysis, we may experience significant deterioration in the mechanical properties. Therefore, PA was dried overnight at 60 °C in a drying furnace.

PA, PE and PP composites were prepared by the addition of SiO_2_–MgO/MWCNT nanocomposites. 50 g of polymer and 0.5 g talc-like oxide coated MWCNT nanocomposite was measured into an internal laboratory mixer (Brabender GNF 106/2). In case of PE and PP, the mixing temperatures were 230 °C and 250 °C for PA. After the heating of the laboratory mixer, half the volume of the polymer granules were added and stirred approximately for 5 min. Afterwards, the second half of the granules and SiO_2_–MgO/MWCNT nanocomposites were added to the polymer blend and the mixture was further stirred for 10 min under constant conditions. The applied rotor speed was kept between 40–50 rpm. A rapid increment of the measured torque was observed at the beginning of the mixing procedure. After a few minutes, the torque was stabilized (4.5 Nm) and remained constant. After the incorporation and mixing, the hot molten compounds were removed from the mixer chamber and the composites were compression moulded (Fontune SRA 100) into 115 × 115 × 1 mm sheet at 250 °C, applying 13 kN compression force. Finally, uniform samples were cut out of these sheets for tensile tests and DSC investigations.

## Results and Discussions

### Investigations of SiO_2_–MgO/MWCNT

**Figure 2:**
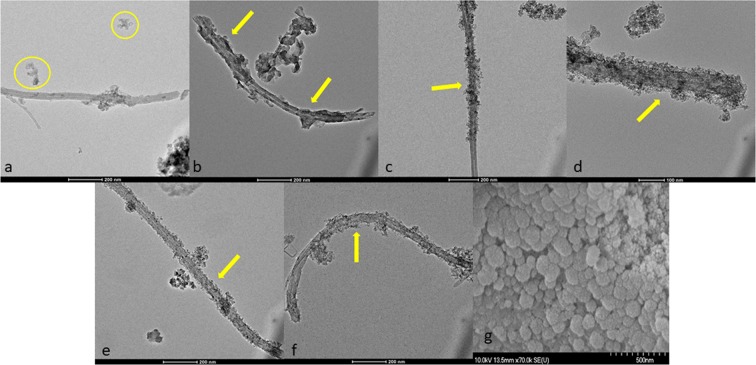
Comparative TEM images of SiO_2_–MgO/MWCNT nanocomposites: (**a**) SiO_2_–MgO/1 wt% MWCNT; (**b**,**c**) SiO_2_–MgO/2 wt% MWCNT; (**d**) SiO_2_–MgO/3 wt% MWCNT; (**e**) SiO_2_–MgO/4 wt% MWCNT; (**f**) SiO_2_–MgO/5 wt% MWCNT, (**g**) SEM image of SiO_2_-MgO particles.

The grayish-black color of samples after the annealing step indicated that the inorganic coating on the surface of MWCNTs was successfully formed. Nanocomposites were investigated with TEM to verify the presence and quality of SiO_2_–MgO coating. Before TEM investigation nanocomposites were pulverized into fine powder in an agate mortar and were sonicated using ultrasonic bath for an extended period of time (15 min). Despite these radical sample pre-treatment, nanoparticles were still detectable on the surface of MWCNTs. Figure 2 shows the micrographs of nanocomposites with different weight ratio of MWCNT. In the samples containing 1 wt% MWCNT mainly nanoparticles separated from the MWCNTs were observed (Fig. 2a). As it can be seen in Fig. 2b–f, with increasing MWCNT content the coverage of their surface seems more homogeneous. The investigation verified that coverage on the surface is quite similar to each other, no significant differences were observed in the quality of coating and evidently, every sample contains separated nanoparticles from the MWCNTs. Investigations revealed that the oxide nanoparticles are in the size range of 10–70 nm and the thickness of the coating is approximately 5–10 nm.

Afterwards, a reference sample of SiO_2_–MgO was investigated by SEM to gather more data about the shape and size of the particles. In Fig. 2g it can be clearly observed that the reference sample contains small particles, which form bigger spherical particles. Based on the SEM studies, the average diameter of these spherical particles is 75.60 nm ± 29.55.

**Figure 3:**
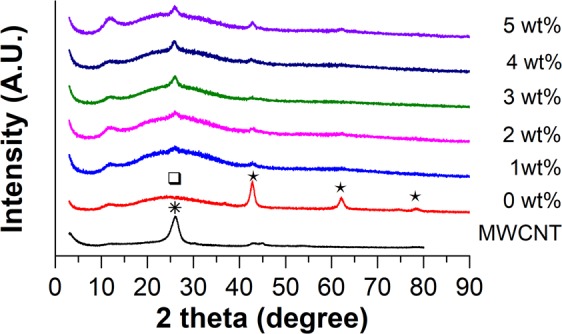
Comparative XRD diffractograms of SiO_2_–MgO/MWCNT nanocomposites.

Figure 3 shows the diffraction patterns of each SiO_2_–MgO/MWCNT nanocomposites (label of the nanocomposites based on their MWCNT content). As it can be seen – and discussed further in details – the XRD measurements confirmed the results of TEM investigations. In the XRD diffraction of reference sample, four characteristics reflections can be observed: the wide reflection at 2θ = 25° suggests an amorphous characteristic of SiO_2_ (marked with ❏), which reflection overlaps with those of MWCNTs (marked with ✷ at 2θ = 26.04°). We can also notice that by increasing the amount of MWCNTs, this reflection becomes more prominent. Reflections at 2θ = 42.75°, 62.16° and 78.63° belong to MgO, (200), (220), (222) (marked with ✶), respectively. Another broad reflection can be seen at 2θ = 11.63°, which could not be identified unequivocally, since phyllosilicates have different variations, which are rather similar to each other. Therefore, this reflection might belong to chrysotile, enstatite or talc.

**Figure 4:**
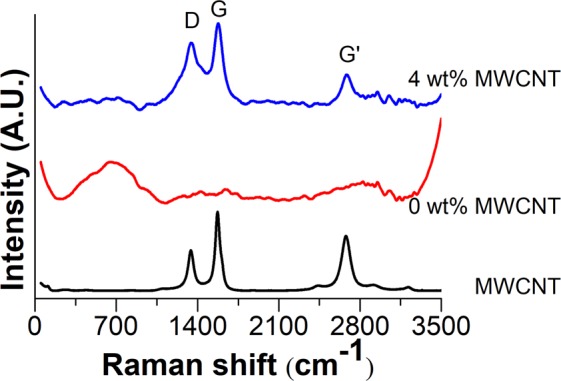
Raman spectra of SiO_2_–MgO/MWCNT nanocomposites.

Figure 4 shows the Raman spectra of the SiO_2_–MgO/MWCNT nanocomposites. The Raman spectroscopic investigations also confirmed the presence of SiO_2_–MgO mixed oxide coverage in the samples. D, G and G’ peaks are the most important features of the Raman spectra of MWCNTs. The spectrum of SiO_2_–MgO/5 wt% MWCNT shows intensive peaks at 1345 cm^−1^, 1574 cm^−1^ and 2685 cm^−1^ which correspond to the D, G, and G’ modes. The Raman active modes of the prepared oxide coating can be observed at 662 cm^−1^, 927 cm^−1^, 1281 cm^−1^, 1428 cm^−1^, 1645 cm^−1^, 2953 cm^−1^, and 3053 cm^−1^. Several materials having lamellar structure possess Raman active peaks at around these Raman shifts. Based on these Raman shifts in connection with XRD diffractograms it can be concluded that our samples are coated by disordered lamellar structure. In case of nanocomposites, the interfacial interaction between the MWCNTs and coating particles is crucial fact for the future applications. In the spectra of MWCNTs the changes of G and D peaks may give information about this. Previous experiments proved the chemical connection between the inorganic layer and MWCNTs^48,49^. The ratio of D and G peaks can give information about the graphitic trait. The lower the ratio, the better graphitic characteristics are possessed by the MWCNTs. This D/G ratio of the pure MWCNT sample was 0.51, while in the case of the SiO_2_–MgO/MWCNT nanocomposites the ratio was between 0.57 and 0.75; the presence of an interaction between the MWCNTs and the oxide coating can also be interpreted indirectly.

XRD and Raman methods confirmed the presence of the inorganic layer on the surface of MWCNTs but they do not give any information about the chemical bonding between the inorganic layer and MWCNTs. In our research group, the nature of connection between MWCNTs and inorganic particles was investigated earlier by Fourier-transform infrared spectroscopy method as well and the formation of chemical bonding between MWCNTs and inorganic layer was proved. This results can be adopted with certainty in case of our samples^50^.

### Investigations of coated MWCNT/polymer composites

After the tensile tests, we investigated the fracture surface of the polymer by SEM. The samples were sputtered with a gold – palladium coating to improve the conductivity of the samples. Creating a conductive metal layer on the composites inhibits charging, and improves secondary electron signal, which is required for topographic examination.

**Figure 5:**
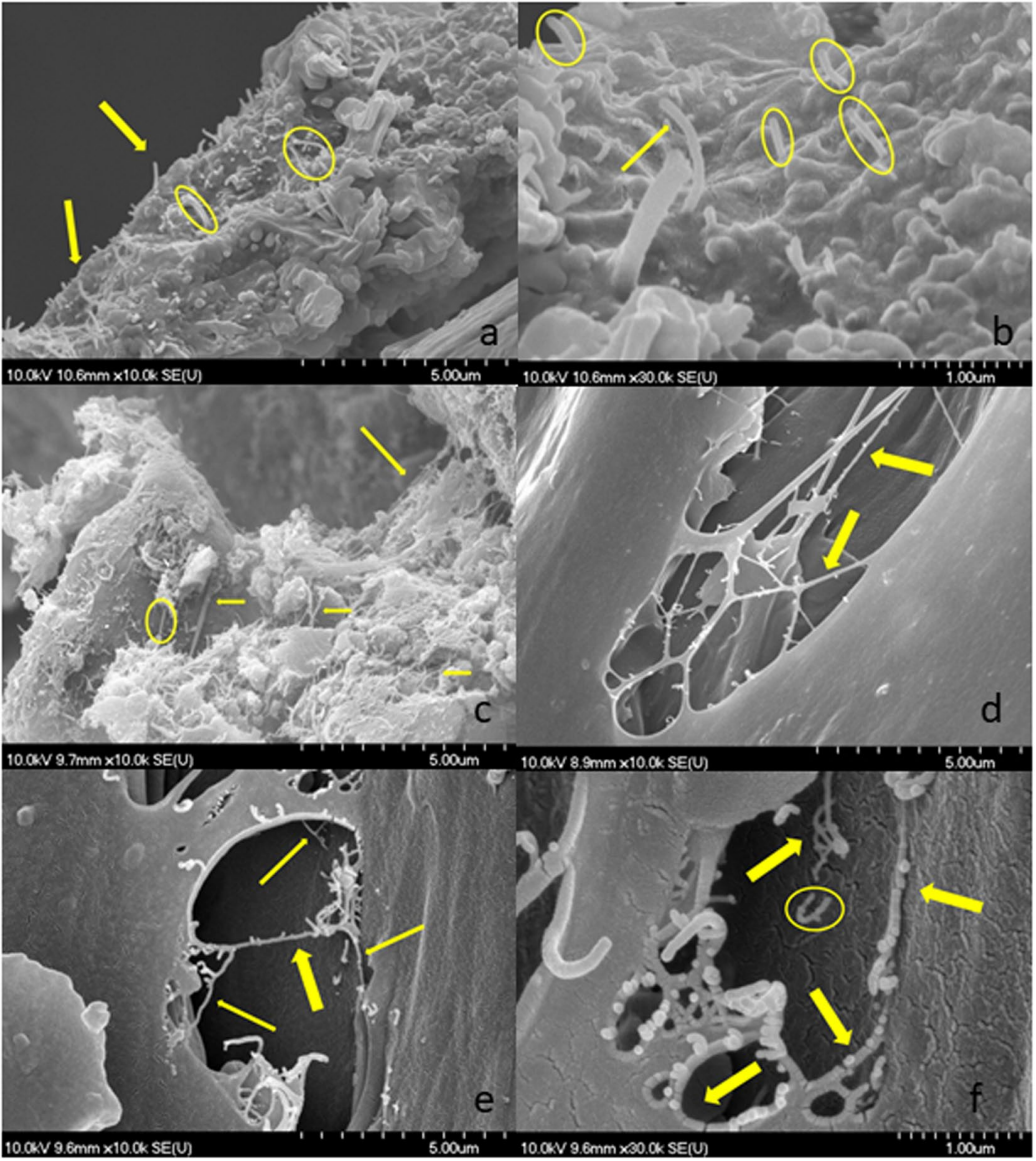
SEM images of different types of SiO_2_–MgO/MWCNT_polymer composites; (**a**,**b**) SiO_2_–MgO/3 wt% MWCNT_PE composite, (**c**) SiO_2_–MgO/4 wt% MWCNT_PE, (**d**) SiO_2_–MgO/4 wt% MWCNT_PA, (**e**,**f**) SiO_2_–MgO/1 wt% MWCNT_PP.

Figure 5 represents the SEM images of SiO_2_–MgO/MWCNT nanocomposites incorporated in different types of polymer matrices. At the fractured sites, coated MWCNTs can be observed, which further supports the success of a durable MWCNT coating.

**Figure 6:**
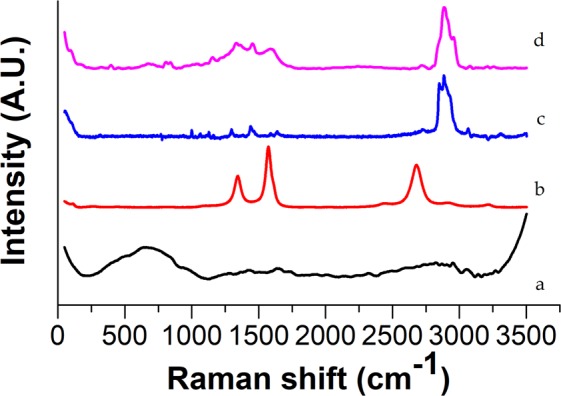
Raman spectra of (**a**) reference SiO_2_–MgO, (**b**) MWCNT, (**c**) neat PA, (**d**) SiO_2_–MgO/4 wt% MWCNT_PA.

**Figure 7:**
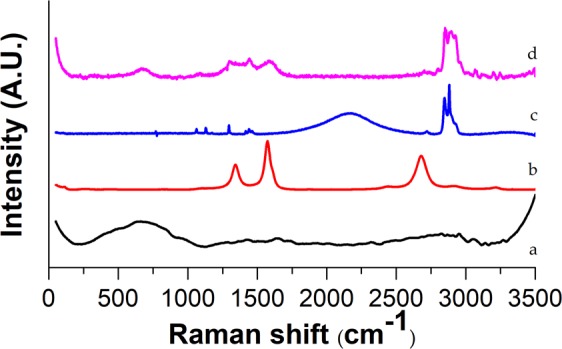
Raman spectra of (**a**) reference SiO_2_–MgO, (**b**) MWCNT, (**c**) neat PE, (**d**) SiO_2_–MgO/1 wt% MWCNT_PE.

Raman spectroscopy confirmed the presence of coated MWCNTs in the polymer matrices. As it can be seen in Figs 6 and 7, the most prominent peaks of the polymer (2896 cm^−1^) and filler materials (665 cm^−1^, 1334 cm^−1^, 1582 cm^−1^ and 2671 cm^−1^) are identifiable in the spectra of SiO_2_–MgO/MWCNT polymer composites.

**Figure 8:**
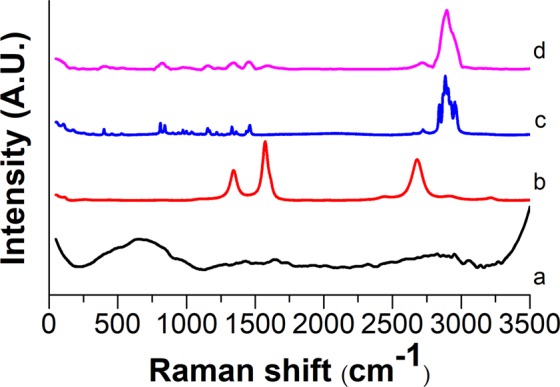
Raman spectra of (**a**) reference SiO_2_–MgO, (**b**) MWCNT, (**c**) neat PP, (**d**) SiO_2_–MgO/4 wt% MWCNT_PP.

**Figure 9:**
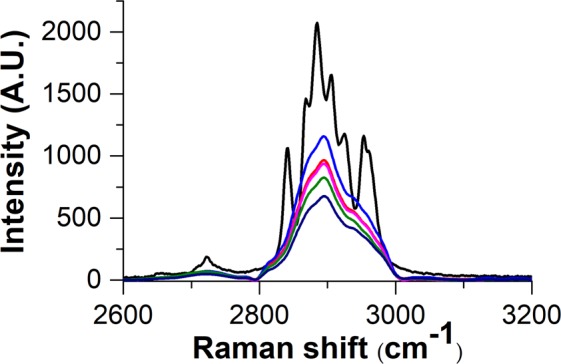
Enlarged Raman spectra of SiO_2_–MgO/MWCNT_PP composites: (black line) PP, (red line) SiO_2_–MgO/1 wt% MWCNT_PP, (blue line) SiO_2_–MgO/2 wt% MWCNT_PP, (purple line) SiO_2_–MgO/3 wt% MWCNT_PP, (green line) SiO_2_–MgO/4 wt% MWCNT_PP, (dark blue line) SiO_2_–MgO/5 wt% MWCNT_PP.

The bands of SiO_2_-MgO and MWCNTs cannot be identified unambiguously in the full range of the spectra (Fig. 8). However, the 2600–3200 cm^−1^ range of spectra (Fig. 9) shows that the SiO_2_–MgO nanoparticles obviously have an effect on the intensity and appearance of the bands of PP. With increasing MWCNT content, the intensity of most characteristic PP peaks proportionally decreased. The changes in band shapes are also well visible, which corresponds to the different optical properties of the composite sample.

**Figure 10:**
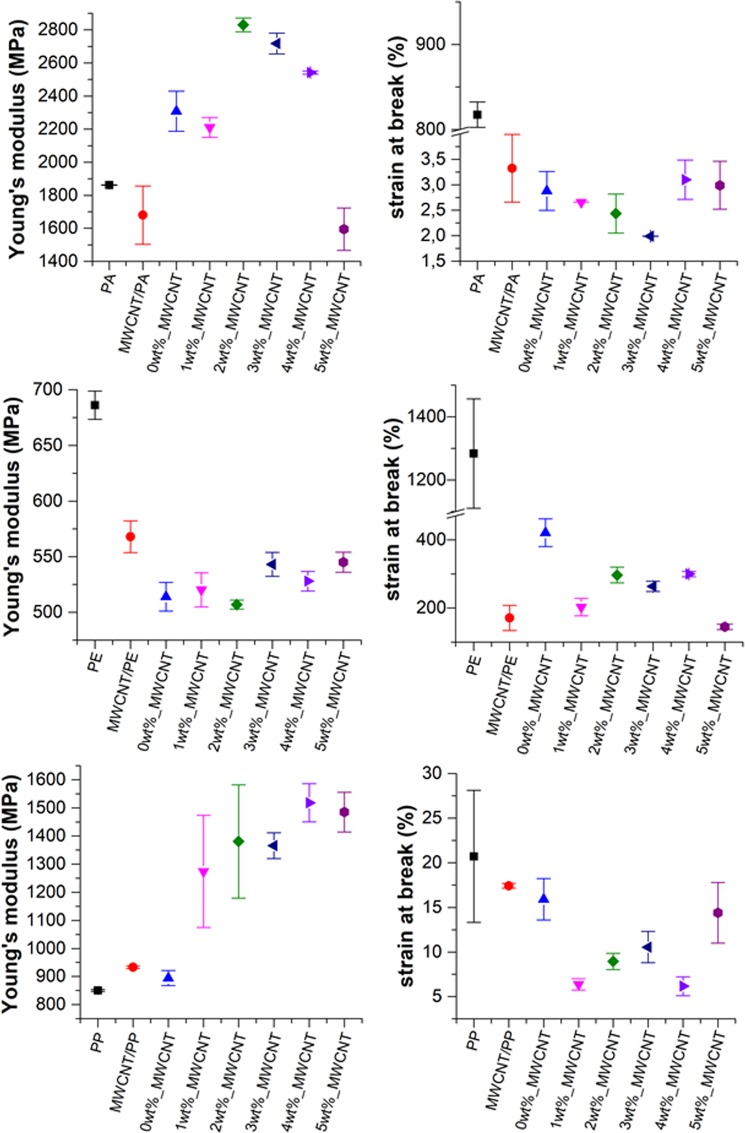
Young’s moduli and strain and break values of neat polymers and composites: (**a**) Young’s moduli of PA composites; (**b**) Young’s moduli of PE composites; (**c**) Young’s moduli of PP composites; symbols are the following: ■ neat polymer, ● MWCNT/polymer, ▲ SiO_2_–MgO, ▼ SiO_2_–MgO/1 wt% MWCNT_polymer, ♦ SiO_2_–MgO/2 wt% MWCNT_polymer, ◄ SiO_2_–MgO/3 wt% MWCNT_polymer, ► SiO_2_–MgO/4 wt% MWCNT_polymer, ⬢ SiO_2_–MgO/5 wt% MWCNT_polymer.

Physical properties of the coated MWCNT/polymer composite samples were examined by tensile tests and DSC. The effects of nanofillers on the mechanical properties are summarized in Table 1 and Fig. 10. By examining these data, we also observed that the mechanical properties of the MWCNT/polymer composite strongly depend on the shape of the nanofiller, the concentration of MWCNT, and the interaction between the filler material and the polymer matrix^51,52^.

**Table 1:** Young’s moduli (Et) and strain at break (ε_B_) of neat polymers and polymer composites.

Specimen	PA		PE		PP	
	Et (GPa)	εB (%)	Et (GPa)	εB (%)	Et (GPa)	εB (%)
polymer without filler	1.86 ± 0.00	817.29 ± 14.90	0.69 ± 0.01	1283.56 ± 173	0.85 ± 0.00	20.72 ± 7.40
MWCNT	1.68 ± 0.18	3.32 ± 0.65	0.57 ± 0.14	170.55 ± 36.68	0.93 ± 0.00	17.43 ± 0.26
SiO_2_-MgO	2.31 ± 0.12	2.88 ± 0.38	0.51 ± 0.01	420.99 ± 40.45	0.89 ± 0.03	15.91 ± 2.32
SiO_2_-MgO/ 1 wt% MWCNT	2.21 ± 0.06	2.65 ± 0.00	0.52 ± 0.02	202.42 ± 25.54	1.27 ± 0.20	6.35 ± 0.64
SiO_2_-MgO/ 2 wt% MWCNT	2.83 ± 0.04	2.44 ± 0.39	0.51 ± 0.00	296.86 ± 23.11	1.38 ± 0.20	8.95 ± 0.91
SiO_2_-MgO/ 3 wt% MWCNT	2.72 ± 0.06	1.99 ± 0.00	0.54 ± 0.01	263.15 ± 15.10	1.37 ± 0.05	10.55 ± 1.77
SiO_2_-MgO/ 4 wt% MWCNT	2.54 ± 0.01	3.10 ± 0.38	0.53 ± 0.01	299.85 ± 7.61	1.52 ± 0.07	6.15 ± 1.06
SiO_2_-MgO/ 5 wt% MWCNT	1.59 ± 0.1	2.99 ± 0.47	0.55 ± 0.01	144.64 ± 7.80	1.49 ± 0.07	14.4 ± 3.39

**Figure 11:**
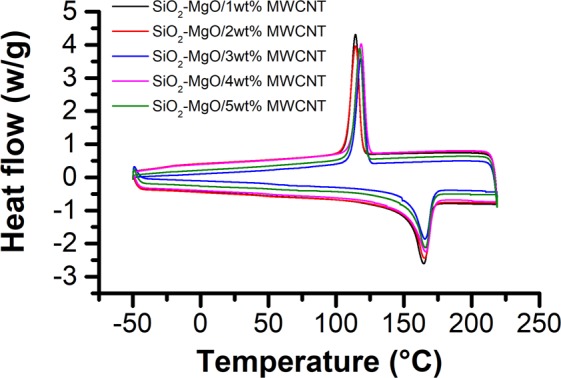
DSC curves of SiO_2_–MgO/MWCNT_PP composites.

As it can be seen in Fig. 10a, the Young’s modulus of the MWCNT/PA nanocomposite was lower than that of the neat polymer This observation can be explained by the tendency of MWCNTs to form bundles because of the strong Van der Waals interaction between the MWCNTs. However, coated MWCNTs provided a significant increase in the mechanical properties of composites. SiO_2_–MgO nanoparticles on the surface of MWCNTs decreased the aggregation tendency of MWCNTs. The sample containing SiO_2_–MgO/5 wt% MWCNT has a much lower Young’s modulus. This observation can be explained with the presence of the agglomerated nanoparticles. It is important to mention that with electron microscopy we can investigate only a small amount of nanocomposite. SiO_2_–MgO/MWCNT sample can contain uncovered MWCNT and also free inorganic nanoparticles which can agglomerate and hereby negatively influence the mechanical properties of the polymer matrix. In case of each PE composite samples, the Young’s moduli were lower than the Young’s modulus of the neat polymer (Fig. 10b). Previous publications already investigated widely the effects of particle size^53–55^, particle-matrix interaction on the mechanical properties of composites^56–58^. The unfavorable effects can be explained by the weak interaction between the filler material and the matrix. Due to the lack of suitable interaction between the filler material and the polymer, the particles tend to agglomerate, and their movement causes vacancies in the matrix. These vacancies may set up deformations in the PE matrix worsening the mechanical properties. By increasing the MWCNT content, this effect is reduced because of the combined effect of inorganic nanoparticles and the fiber-like MWCNTs. Figure 11c illustrates the results of the tensile tests of PP composites. It can be observed that nanoparticles positively influenced the mechanical properties of the matrix. The Young’s modulus of the neat PP was 850 MPa, which increased to 1555 MPa as an effect of SiO_2_–MgO/MWCNT nanoparticles. The wide error bar in the case of the samples containing SiO_2_–MgO/1 wt% MWCNT and SiO_2_–MgO/2 wt% MWCNT can be explained by the fact that only a small amount of filler material was incorporated into a large amount of polymer, and thus the homogenous dispersion in the matrix is more difficult to achieve. Based on the TEM images, it can be concluded that the separated SiO_2_–MgO nanoparticles in the samples can also impair the mechanical properties. In the SEM picture already seen **(**Fig. 5e) MWCNTs connect the broken parts of the polymer and the tensile tests showed strengthened properties, from which we can conclude that these SiO_2_-MgO/MWCNT nanoparticles – similar to the case of reinforced concrete – keep the polymer parts together.

The addition of SiO_2_–MgO/MWCNT nanofillers into the polymer matrices also influenced the strain at break values. Based on the results illustrated in Fig. 10a–c, it could be generally inferred that the strain at break values of the unfilled polymers were higher than that of the filled polymer composites. Whilst investigating the shape of the fillers, we can see that the application of a fiber-shaped reinforcement material resulted in a higher strain at break value, which verified the fact that different types of filler materials have dissimilar effects on the mechanical properties. As Fig. 10a represents, SiO_2_-MgO/MWCNT fillers make the polymer matrix more rigid, but by increasing the content of MWCNT, an even higher strain at break value can be achieved. It can be clarified that contrary to fiber reinforcement, SiO_2_–MgO fillers have irregular shape, and thus nanoparticles cannot properly transmit stress from the matrix^59^. In Fig. 10b,c, higher strain at break values can be observed, which can be explained by the lower dispersity of nanoparticles. Previously, patterns were investigated, in which nanoparticles did not accumulate and for this reason resulted higher value.Summarizing the results of tensile test, it can be concluded that SiO_2_-MgO/MWCNT nanocomposites changed the rigidity of polymers. This fact confirmed the good dispersity of the MWCNTs in the polymer matrices.

Figure 11 shows the typical DSC curves of SiO_2_–MgO/MWCNT_PP nanocomposites. The endothermic peak at around 160 °C represents the melting temperature of PP. From Fig. 11 it can be obviously seen that the filler contentdid not influence significantly the melting temperature at melting point. The melting temperature, crystallization peak temperature, heat of fusion and degree of crystallinity of the nanocomposites are summarized in Table 2. As represented in Table 2, the melting and crystallization temperatures of the polymer nanocomposites did not change significantly as the MWCNT content increased. We can also see that the crystallinity percentage of SiO_2_–MgO/MWCNT_PP decreased slightly with increasing MWCNT content, but no significant changes could be observed. This observation can be explained by the nucleating activity of MWCNT networks, which can influence the mobility of polymer chains and slow down the crystal growth. This observation correlates with others, which demonstrated that a competition between nucleation and confinement appears. The curves of PA and PE composites represented identical results, thus they arenot reported in this study.

**Table 2:** Crystallization and melting properties of SiO_2_–MgO/MWCNT_PP polymers.

Specimen	T_c_ (°C)	Tm (°C)^a^	ΔH (J/g)^a^	X_1_^a^	Tm (°C)^b^	ΔH (J/g)^b^	X_2_^b^
SiO_2_–MgO/1 wt% MWCNT	119.03	153.90	87.24	41.75	153.85	86.10	41.20
SiO_2_–MgO/2 wt% MWCNT	112.50	154.21	83.98	40.20	152.95	82.90	39.67
SiO_2_–MgO/3 wt% MWCNT	123.05	153.39	69.26	33.15	155.17	76.21	36.47
SiO_2_–MgO/4 wt% MWCNT	123.24	151.96	86.26	41.29	155.44	85.75	41.05
SiO_2_–MgO/5 wt% MWCNT	121.77	154.73	77.24	36.98	156.05	78.12	37.40

Tc crystallization temperature, Tm melting temperature, ΔH heat of fusion, X percent crystallinity after first heating (a) and second heating (b).

## Conclusions

This article presented the synthesis and investigation of SiO_2_–MgO coated MWCNT nanocomposites and their polymer composites. Coated MWCNT nanocomposites were prepared by sol-gel technique with different multiwalled carbon nanotube content. TEM, XRD and Raman investigations proved that the surface of MWCNTs was successfully coated with inorganic nanoparticles, and after annealing, the desired SiO_2_–MgO layer was formed. Based on the tensile test results, we conclude that in the case of PA and PP matrix, the nanomaterials positively influenced the moduli. We observed that the shape of the fillers can influence the strain at break values. In general fiber-shaped reinforcement material can cause higher strain at break values. In case of our samples aggregated nanoparticles on the surface of MWCNTs caused reduced values. Our results showed that the physical properties of polymers have changed and increased their brittleness. This observation suggests that the dispersion of MWCNTs increased in the polymer matrices. This verifies our proposition that different types of filler materials have dissimilar effects on the mechanical properties. Whilst investigating the DSC curves, significant changes could not be observed in the thermal properties of the prepared polymers.
